# Role of the hypoxia response pathway in lens formation during embryonic development of *Xenopus laevis*^☆^

**DOI:** 10.1016/j.fob.2013.10.006

**Published:** 2013-10-23

**Authors:** Kazunobu Baba, Taichi Muraguchi, Susumu Imaoka

**Affiliations:** Research Center for Environmental Bioscience, School of Science and Technology, Kwansei Gakuin University, 2-1 Gakuen, Sanda, Hyogo, Japan; Department of Biosciece, School of Science and Technology, Kwansei Gakuin University, 2-1 Gakuen, Sanda, Hyogo, Japan

**Keywords:** Siah2, HIF-1α, EMT, Lens formation, Siah2, seven in absentia homolog 2, PHDs, prolyl hydroxylase domains, HIF-1α, hypoxia-inducible factor-1α, pVHL, von Hippel–Lindau tumor suppressor protein, VEGF, vascular endothelial growth factor, PLE, presumptive lens ectoderm, EMT, endothelial mesenchymal transition, *E. coli*, *Escherichia coli*, PCR, polymerase chain reaction, pBS, pBluescriptII+, MBS, Modified Birth’s Solution, NBT, nitro-blue tetrazolium chloride, SDS, sodium dodecylsulfate

## Abstract

The RING finger ubiquitin ligase seven in absentia homolog 2 (Siah2) was identified in the R7 photoreceptor cells of *Drosophila melanogaster*, and it regulates the stability of prolyl hydroxylase domains (PHDs), with a concomitant effect on HIF-1α availability in the hypoxia response pathway. We previously reported that the hypoxia response pathway contributes to eye development during the embryonic development of *Xenopus laevis*. In this paper, the role of Siah2-mediated hypoxia response pathway in eye development of *X. laevis* embryos was further characterized. *Xenopus Siah2* (xSiah2) mRNA was detected in lens tissue and xSiah2 overexpression caused a thickened lens placode, leading to loss of the optic lens. In embryos overexpressing xSiah2, lens marker gene transcription was reduced, suggesting that xSiah2 contributes to lens formation. xSiah2 overexpression decreased *Xenopus* PHD accumulation and increased *Xenopus* HIF-1α (xHIF-1α) accumulation. xHIF-1α degeneration with resveratrol restored the optical abnormality caused by xSiah2 overexpression, suggesting that the xSiah2-mediated hypoxia response pathway contributes to lens formation. Moreover, xSiah2 overexpression decreased endothelial–mesenchymal transition (EMT)-related Notch signaling-responsive genes transcription during the invasion of the lens placode. Our results suggest that the hypoxia response pathway plays an important role in the regulation of the EMT via the Notch signaling pathway during lens formation.

## Highlights

• The ubiquitin lyase Siah2 regulates HIF-1α availability in the hypoxia response pathway.
• xSiah2 overexpression causes loss of the lens of *Xenopus laevis*.
• *xSiah2* mRNA is detected in the lens placode from embryonic stage 30.
• xHIF-1α overexpression at stage 38 causes loss of the lens of *Xenopus laevis*.
• xSiah2 overexpression suppresses endothelial–mesenchymal transition-related genes.

## 1 Introduction

Siah2 was identified in the R7 photoreceptor cells of *Drosophila melanogaster* [1]. Siah2 functions to target diverse protein substrates for degeneration via ubiquitination. In the hypoxia response pathway, Siah2 mediates efficient ubiquitination to regulate the stability of prolyl hydroxylase (PHD) [2]. The mammalian genome encodes three closely related PHD proteins, designed as PHD1, PHD2, and PHD3. PHD3 interacts with either PHD1 or PHD2, leading to the formation of PHD complexes. Tight regulation of the PHD complex activity and stability affects the availability of hypoxia inducible factor-1α (HIF-1α) [3]. PHD proteins require molecular oxygen to hydroxylate HIF-1α, which in turn becomes a signal for the degeneration of HIF-1α via interaction with the von Hippel–Lindau tumor suppressor protein (pVHL) ubiquitin ligase complex [4]. Available HIF-1α, after the interaction with HIF-1β [5], is a transcription factor responsible for the expression of target genes such as vascular endothelial growth factor (VEGF) gene [6].

The possible involvement of the hypoxia response pathway in the neurogenesis of vertebrates such as mice and frogs has recently been reported. HIF-1α knockout mice show defective angiogenesis as well as abnormal neurogenesis. Overexpression of *Xenopus* Siah2 (xSiah2) in *Xenopus laevis* causes the small eye phenotype [7]. This optical abnormality apparently results from a deficient lens.

Lens tissue is formed during the neurula and tailbud stages of *Xenopus* development. There are four phases of lens formation: (1) presumptive lens ectoderm (PLE) is formed in the surficial layer of the embryo during the neurula stages; (2) interaction between the PLE and anterior neural tube results in PLE thickening and development into a lens placode during the early tailbud stage; (3) the lens placode invaginates and develops into a vesicle through the endothelial mesenchymal transition (EMT): and (4) differentiation into cellular layers occurs [8].

We previously isolated two *Xenopus* PHD (xPHD) proteins, xPHD45 and xPHD28, and characterized them during the embryonic development of *X. Laevis* [9]. In the embryonic development, the co-injection with *xPHD28* mRNA restores the small eye phenotype caused by xSiah2 overexpression, suggesting that xSiah2 contributes to eye development via xPHD. However, the function of the hypoxia response pathway in embryonic sensory organogenesis, including the lens, remains unclear. Given the importance of xSiah2 in the stability of xPHD and consequent *Xenopus* HIF-1α (xHIF-1α) levels, we asked whether the hypoxia response pathway plays a potential role in lens formation.

## 2 Materials and methods

### 2.1 Chemicals and antibodies

Resveratrol was purchased from Sigma (St Louis, MO); MMLV reverse transcriptase from Fermentas (Burlington, Canada); KOD plus DNA polymerase from TOYOBO (Tokyo, Japan); and T3, T7, and SP6 RNA polymerases and Go taq polymerase from Promega (Madison, WI). Antihuman β-actin antibody was purchased from Sigma and horseradish peroxidase-conjugated antirabbit IgG antibody was purchased from Bio-Rad (Hercules, CA). Antixenopus Siah2 antibody was prepared as follows. The first half of xSiah2 were ligated into pQE80L vector (QIAGEN, Hilden, Germany), which allows protein expression in *Escherichia coli* (*E. coli*) strains. xSiah2 peptide was then expressed in *E. coli* DH5α and purified using Ni-NTA agarose (QIAGEN). Antibodies were then raised against human PHD3, xSiah2, and human HIF-1α in rabbits using a previous described method [9,10]. Reaction of the antihuman PHD3, HIF-1α and β-actin antibodies with xPHD, xHIF-1α and *Xenopus* β-actin, respectively, was confirmed. All experiments were conducted in accordance with guidelines on the welfare of experimental animals and with the approval of the Ethics Committee on the use of animals of Kwansei Gakuin University.

### 2.2 Isolation of RNA and RT-PCR analysis

Total RNA extracted from 5 embryos was prepared with Isogen (Nippon gene, Toyama, Japan) according to the manufacturer's instructions. cDNA was synthesized using total RNA (1 μg) in a total volume of 10 μL with MMLV reverse transcriptase according to the manufacturer's instructions as follows: incubation at 25 °C for 15 min and at 42 °C for 60 min followed by heating at 70 °C for 10 min. Polymerase chain reaction (PCR) was performed at 94 °C for 2 min and then for a particular number of cycles of 94 °C for 30 s, 55 °C for 30 s, and 72 °C for 30 s in a reaction mixture containing 10 pmol of each primer, Go taq polymerase, and cDNA (100 ng). Primers, GenBank accession numbers, cycles, and sequences for PCR are shown in Table 1. The PCR products were separated by electrophoresis on a 1% agarose gel, visualized with ethidium bromide staining, and quantified by scanning densitometry using ImageJ software (version 1.36b; National Institutes of Health, Bethesda, MD). The relative mRNA transcript levels were normalized by Histone H4 (Genbank: M21286).

### 2.3 Isolation of xSiah2

cDNA of xSiah2 (GenBank: AF155509) was amplified by PCR. Thirty-five cycles of PCR (94 °C for 30 s, 57 °C for 30 s, and 68 °C for 90 s) were performed using the cDNA obtained from reverse transcription of total RNA from embryos as the template, KOD plus DNA polymerase and corresponding primer pairs. Primer pairs are shown in Table 2: primers 1 and 2 for xSiah2/pBluescriptII+ (pBS), primers 3 and 4 for xSiah2/pCS2+, and primers 3 and 5 for xSiah2/pQE80L. The cDNA of xSiah2 was digested with BamHI and SpeI, BamHI and EcoRI, or BamHI and HindIII and then ligated into pBS (Agilent Technologies, Santa Clara, CA), pCS2+ (RZPD, Berlin, Germany), or pQE80L (Qiagen, Valencia, CA), respectively.

### 2.4 Capped mRNA synthesis and micro-injection

*GFP* and *xSiah2* mRNAs were prepared from GFP/pCS2+ and xSiah2/pCS2+, respectively. After the plasmids were linearized with the restriction enzyme NotI, capped mRNAs were made using a mCAP RNA synthesis kit (Promega) according to the manufacturer's instructions. Synthesized mRNAs (total 2 ng/cell) were injected into each dorsal blastomere at the two-cell stage.

### 2.5 Eggs and embryos of X. laevis

Unfertilized eggs of wild type and albino *X. laevis* (Watanabe Zoushoku, Hyogo, Japan) were obtained by injecting a female with 120 units of human chorionic gonadotropin (Kowa, Tokyo, Japan). The eggs were fertilized with the chestnuts suspended in 1.0× Modified Birth's Solution (MBS) containing 0.5 mM HEPES (pH 7.5), 10 mM NaCl, 0.2 mM KCl, 0.1 mM MgCl_2_, and 0.2 mM CaCl_2_. The chestnuts were surgically isolated from a male. The fertilized embryos were dejellied with 1% sodium thioglycollate and washed with 0.1× MBS several times. The developmental stage of embryos was determined according to Nieuwkoop and Faber's normal table of *X. laevis* [11].

### 2.6 Whole mount in situ hybridization

Thirty albino embryos were fixed in fully dehydrated ethanol. Sense and antisense probes for xSiah2 were prepared from xSiah2/pBS and then linearized with SpeI or BamHI, respectively, and transcribed with T3 or T7 RNA polymerase, respectively, in the presence of digoxygenin UTP (Roche). Hybridized probes were visualized according to the Roche DIG protocol with a minor alteration that 0.45 mL of nitro-blue tetrazolium chloride (NBT) (75 mg/mL in dimethyl formamide) and 3.5 mL of 5-bromo-4-chloro-3′-indolylphosphatase *p*-toluidine salt (BCIP) (Roche) were added to 1 mL of alkaline phosphatase buffer containing 100 mM Tris–HCl (pH 9.5), 100 mM NaCl, 50 mM MgSO_4_, 0.1% Tween 20, and 25 mM levamisole.

### 2.7 Western blotting

Twenty embryos were homogenized in buffer containing 50 mM Tris–HCl (pH 7.4), 1 mM EDTA, 1 mM DTT, 150 mM KCl, and 100 mM PMSF, and then solubilized with sodium dodecylsulfate (SDS). The resulting solution was subjected to SDS–polyacrylamide gel electrophoresis. Proteins were blotted onto a nitrocellulose membrane and reacted with antibodies against human β-actin, human HIF-1α, human PHD, and xSiah2.

### 2.8 Histological analysis

Twenty embryos were fixed in fully dehydrated ethanol and embedded in paraffin. Sagittal sections were cut 10 μm thick and stained with hematoxylin and eosin.

### 2.9 Statistical analysis

All data are reported as mean ± SD. Statistical analysis of the data was performed by one-way ANOVA. Significance was determined by ANOVA followed by Fisher's protected least significant difference.

## 3 Results

### 3.1 Localization of xSiah2 mRNA during development of X. laevis

**Fig. 1:**
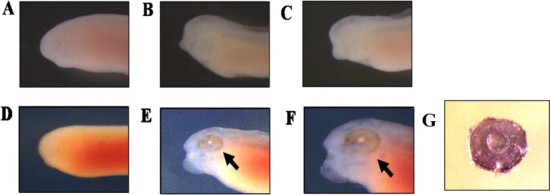
Accumulation of *xSiah2* mRNA during the development of *Xenopus laevis*. (A–G) The accumulation pattern of *xSiah2* mRNA during tailbud stages was investigated by whole mount *in situ* hybridization. Images (A–C) show anterior views of the head of albino embryos hybridized with the sense probe at st. 24, 30, and 38, respectively. Images (D–F) show anterior views of the head of albino embryos hybridized with the antisense probe at st. 24, 30, and 38, respectively. Arrows indicate the lens tissue. Image (G) shows a single eye surgically detached from the embryo and hybridized with the antisense probe at st. 38.

We previously suggested that Siah2 contributes to eye formation, which initiates at st. 24 during the development of *X. laevis* [12]. Hence, albino embryos of *X. laevis* were grown until st. 24 (early tailbud stage), 30 (middle tailbud stage), and 38 (later tailbud stage) and harvested at each stage. The accumulation pattern of *xSiah2* mRNA in these embryos was investigated by whole *in situ* hybridization (Fig. 1A–G). *xSiah2* mRNA was not detected anywhere at st. 24 (Fig. 1D), and was detected in the lens placode at st. 30 (Fig. 1E) and 38 (Fig. 1F and G). Accordingly, we focused on the role of Siah2 in lens formation during development of *X. laevis*.

### 3.2 Contribution of xSiah2 to lens formation

**Fig. 2:**
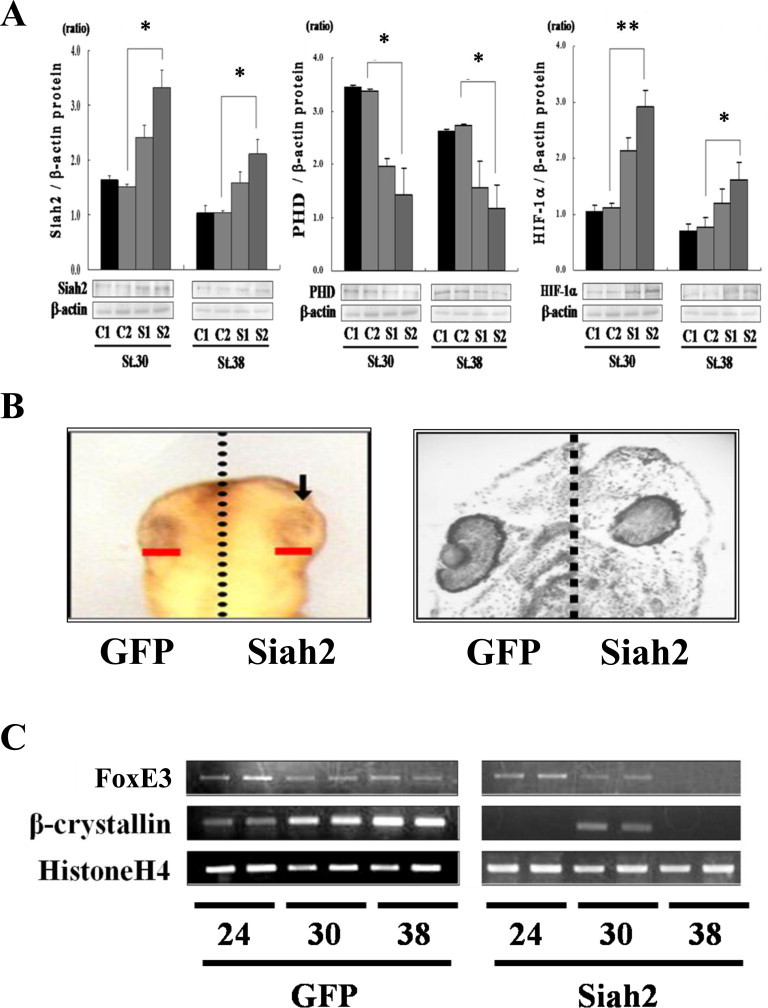
Effects of *xSiah2* mRNA injection on the accumulation of xSiah2, xPHD, and xHIF-1α, eye phenotype, and lens marker transcription. (A) In C1 embryos, synthesized mRNA was not injected; in C2 embryos, *GFP* mRNA was injected into both dorsal blastomeres at the two-cell stage; in S1 embryos, *GFP* mRNA was injected into one dorsal blastomere, and *Siah2* mRNA into another dorsal blastomere at the two-cell stage; and in S2 embryos, *xSiah2* mRNA was injected into both dorsal blastomeres at the two-cell stage. The expression levels of xSiah2, xPHD, and xHIF-1α at st. 30 and 38 were investigated in these embryos by western blotting. The values in the graphs represent the mean ± SD of three experiments. *p < 0.05, **p < 0.01 vs. C2 embryos (B) *GFP* or *xSiah2* mRNA was injected into each dorsal blastomere at the two-cell stage. Comparison of anterior views of the head between the sides of the same embryo injected with *GFP* mRNA (GFP) and *xSiah2* mRNA (Siah2) at st. 30. Arrow indicates the thickened lens ectoderm. Histological difference between the sides of the same embryo injected with *GFP* mRNA (GFP) and *xSiah2* mRNA (Siah2) at st. 38. (C) *GFP* (GFP) or *xSiah2* mRNA (Siah2) was injected into both dorsal blastomeres at the two-cell stage. The transcriptional levels of *FoxE3* and *β-crystallin* at st. 24, 30, and 38 were investigated in these embryos by RT-PCR.

At the two-cell stage, *xSiah2* mRNA was injected into either one dorsal blastomere (S1 treatment group) or both dorsal blastomeres (S2 treatment group). The effect of xSiah2 overexpression on the level of xPHD and xHIF-1α accumulations at st. 30 and 38 was then investigated by western blotting (Fig. 2A). In the S2 treatment group, the level of xSiah2 and xHIF-1α proteins was significantly increased at st. 30 and 38; xPHD was conversely decreased, suggesting that xSiah2 overexpression induced the degeneration of xPHD with concomitant effect on the enrichment of available xHIF-1α.

Next, to investigate the effect of xSiah2 overexpression on lens formation, the organogenesis of eyes in *X. laevis* embryos was observed (Fig. 2B, left side). *GFP* or *xSiah2* mRNA was injected into each dorsal blastomere at the two-cell stage, and the embryos were harvested at st. 30. The side of embryo injected with *GFP* mRNA developed a normal dorsal head region phenotype. On the side of the embryos injected with *xSiah2* mRNA, a thickened lens ectoderm was observed. Next, these embryos were grown to st. 38 and then harvested for histological analysis (Fig. 2B, right side). While the side of embryo injected with *GFP* mRNA had normal eyes, the side injected with *xSiah2* mRNA demonstrated loss of the lens as well as thickened lens ectoderm.

Next, *xSiah2* mRNA was injected into both dorsal blastomeres at the two-cell stage, and the effect of xSiah2 overexpression on the transcriptional levels of the lens marker genes, *FoxE3* (Genbank: BC169818) and *β-crystallin* (Genbank: BC084735) mRNAs, at st. 30 and 38 were then investigated by RT-PCR (Fig. 2C). FoxE3 represses differentiation in the undifferentiated lens ectoderm, and β-crystallin is expressed in the differentiated lens ectoderm [12]. While the transcriptional level of *FoxE3* mRNA was not affected by xSiah2 overexpression at st. 24, it was decreased at st. 30 and 38. The transcriptional level of *β-crystallin* mRNA was decreased at all stages, indicating that xSiah2 contributes to lens formation and, in particular, to the differentiation of lens endothelial cells.

### 3.3 Contribution of the Siah2-mediated hypoxia response pathway to lens formation

**Fig. 3:**
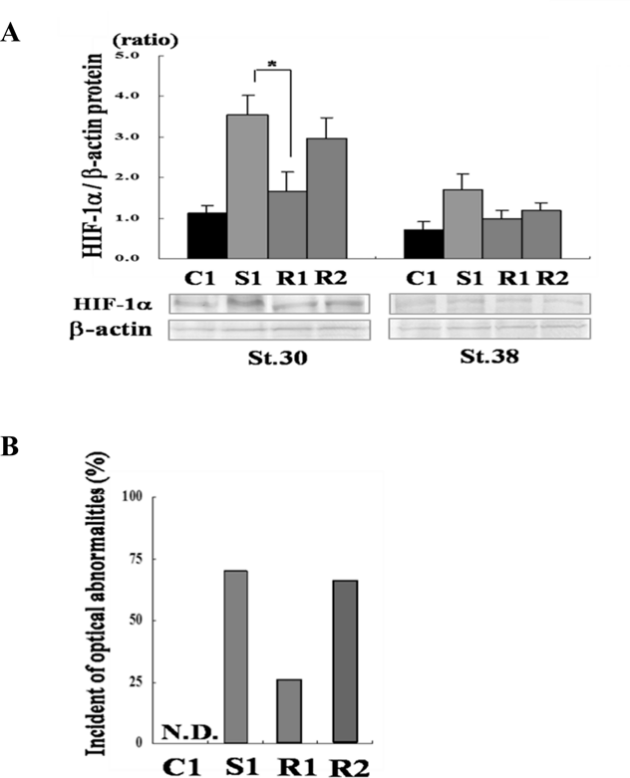
Effect of resveratrol on the small eye phenotype. (A and B) In the C1 treatment group, *GFP* mRNA was injected into both dorsal blastomeres at the two-cell stage. In the S1, R1, and R2 treatment groups, *xSiah2* mRNA was injected into both dorsal blastomeres at the two-cell stage. Embryos were exposed to resveratrol (40 μM) from st. 12 to 38 in R1 treatment group and from st. 22 to 38 in the R2 treatment group. (A) The expression levels of xHIF-1α at st. 30 and 38 were investigated in these embryos by western blotting. The values in the graph represent the mean ± SD of three experiments. *p < 0.05 vs. S1 treatment group. (B) Percentages of embryos with optical malformations in each treatment group at st. 38. N.D., not detected.

Siah2 functions to target diverse protein substrates for degeneration via ubiquitination. We previously found that co-injection with xPHD restores the optical abnormalities caused by xSiah2 overexpression. This suggests that xPHD is a target substrate of xSiah2 during eye development. Accordingly, the role of xSiah2-mediated hypoxia response pathway in lens formation was investigated. *GFP* or *xSiah2* mRNA was injected into both dorsal blastomeres of embryos at the two-cell stage; *xSiah2* mRNA-injected embryos were then exposed to resveratrol, an inhibitor of HIF-1α [13]. Embryos were treated with resveratrol from st. 12 to 38 in R1 treatment group, and from st. 22 to 38 in R2 treatment group. The level of xHIF-1α at st. 30 or 38 in each treatment group was investigated in these embryos by western blotting (Fig. 3A). Treatment with resveratrol from st. 12 to 38 restored xHIF-1α expression to normal at st. 30. Exposure to resveratrol from st. 22 to 38 did not affect the level of xHIF-1α expression at st. 30 or st. 38. Next, the organogenesis of the eyes was evaluated in these groups (Fig. 3B). The percentage of embryos with optical malformations caused by *xSiah2* mRNA injection was reduced by treatment with resveratrol from st. 12 to 38, but not from st. 22 to 38, suggesting that xSiah2 may contribute to lens formation via xHIF-1α.

### 3.4 Disruption of the Notch signaling pathway by Siah2 overexpression

**Fig. 4:**
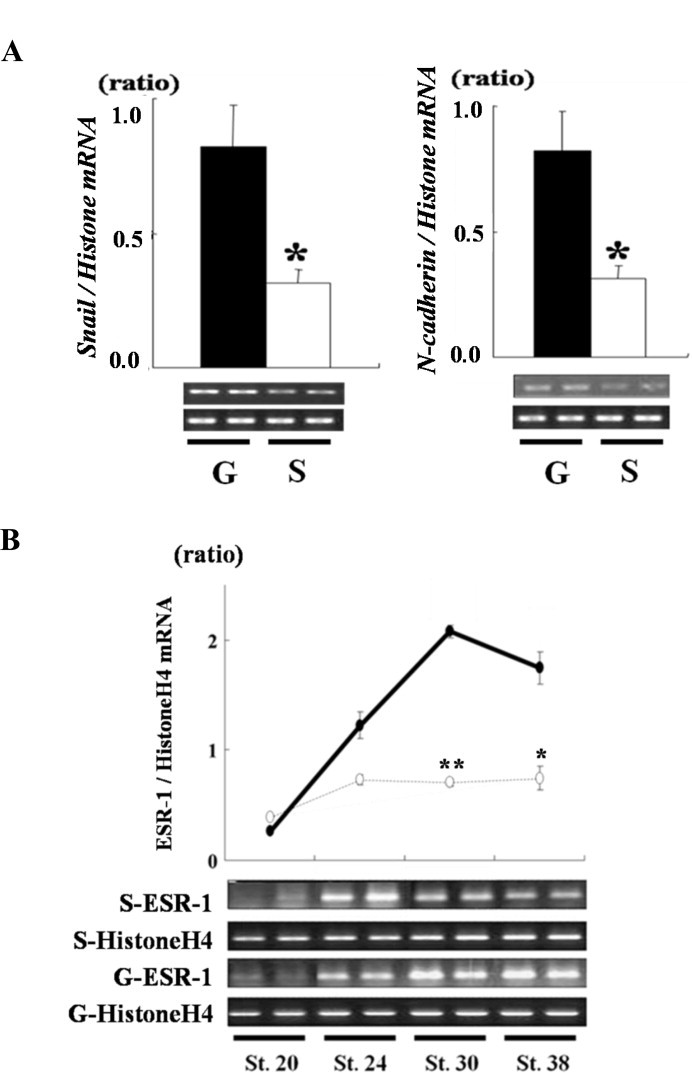
Effect of *xSiah2* mRNA injection on transcriptional levels of Notch signaling-responsive genes. (A and B) *GFP* or *xSiah2* mRNA was injected into both dorsal blastomeres at the two-cell stage. (A) The transcriptional levels of *Snail-1* and *N-cadherin* mRNAs at st. 30 were investigated in the embryos by RT-PCR. Black and white bars indicate the transcriptional levels in *GFP* mRNA injected embryos (G) and *xSiah2* mRNA injected embryos (S), respectively. The values in the graphs represent the means ± SD of three experiments. *p < 0.05 vs. *GFP* mRNA-injected embryos (B) The transcriptional levels of *ESR-1* mRNA at st. 20, 24, 30, and 38 were investigated by RT-PCR. Closed and open circles indicate the transcriptional levels of *ESR-1* mRNA in *GFP* mRNA-injected embryos and *xSiah2* mRNA-injected embryos, respectively. The values in the graph represent the means ± SD of three experiments. *p < 0.05, **p < 0.01 vs. *GFP* mRNA-injected embryos.

An EMT process is involved in the initial step of lens vesicle formation [14]. The EMT converts epithelial cells with a nonmotile morphology into migratory cells that can invade other tissues. The EMT is accompanied by changes in the expression of specific genes, such as *Snail-1* and *N-cadherin* [15,16]. Snail-1 functions to induce *N-cadherin* mRNA transcription during the EMT of endothelial cells. Both *Snail-1* and *ESR-1* mRNAs are downstream factors in Notch signaling [15,17]. To investigate the effect of xSiah2 overexpression on the EMT, the transcriptional levels of the EMT-related genes, *N-cadherin* (Genbank: X57675)*, Snail-1* (Genbank: BC056857)*,* and *ESR-1* (Genbank: AF383157) were investigated at st. 30 using RT-PCR (Fig. 4A and B). XSiah2 overexpression repressed expression of all the EMT-related genes, *Snail-1*, *N-cadherin,* (Fig. 4A) and *ESR-1* (Fig. 4B) at st. 30. These results indicate that xSiah2 overexpression inhibited the activity of Notch signaling, leading to EMT-mediated lens vesicle formation.

## 4 Discussion

*xSiah2* mRNA was not detected during the development of the PLE into the lens placode but was detected while the lens placode was invaginating and differentiating. This result suggests that xSiah2 participates in lens vesicle formation and/or differentiation into cellular layers rather than PLE formation and thickening of the lens placode. FoxE3 functions to thicken the lens placode; however, xSiah2 overexpression did not affect the transcription of *FoxE3* mRNA at st. 24, and also, *xSiah2* mRNA was not detected anywhere at st. 24, thereby strongly confirming that xSiah2 did not participate in the thickening of the lens placode. xSiah2 overexpression causes thickening of lens ectoderm even at st. 30. This morphological difference is probably due to defective invagination of the lens vesicle caused by xSiah2 overexpression, indicating that xSiah2 contributes to the invagination of the lens vesicle rather than the differentiation of the cellular layers.

xSiah2 overexpression induced a decrease in xPHD expression. In addition, xPHD overexpression by co-injection with xSiah2 restored the absence of lens formation caused by xSiah2 overexpression, suggesting that xPHD is the substrate of xSiah2 and that xSiah2 participates in lens formation via xPHD *in vivo*. In the *Xenopus* hypoxic response pathway in lens endothelial cells, xPHD interacts with another xPHD, which could be xPHD1. The Xenopus homolog of human PHD2 is known, but this is unlikely to be involved in lens formation as it is not found in the lens region (data not shown). At low oxygen concentrations *in vitro*, PHDs exhibit 10% and 50% of their maximum hydroxylase activity toward their substrates, including HIF-1α [18]. During lens vesicle formation, the active degeneration of xPHDs by xSiah2 induced the suppression of xPHD hydroxylase activity more efficiently than that by regulation of oxygen alone. The optical malformation was again seen when the xHIF-1α overexpression induced by xSiah2 injection was reversed by resveratrol treatment at st. 30 but not at st. 38. This result suggests that xSiah2 participates in lens formation via xHIF-1α at st. 30, when the lens vesicle is being formed, indicating that xSiah2 participates in lens vesicle formation via xHIF-1α.

In this study, xSiah2 overexpression caused a decrease in the expression of EMT-related genes such as *Snail-1*, *N-cadherin* and *ESR-1*. This result suggests that the disruption of EMT by xSiah2 overexpression inhibited lens vesicle formation, leading to the lens defect. The xSiah2-mediated hypoxia response pathway thus participates in the EMT. Snail-1, ESR-1 and FoxE3 are directly up-regulated by Notch signaling. However, xSiah2 overexpression suppressed the transcription of *Snail-1* and *ESR-1* mRNAs at st. 30, but not that of *FoxE3* mRNA at st. 24. Notch signaling is the communication system between cells. During lens formation, some Notch signaling pathways are activated independently. Between retinal progenitor cells in the optic cup and lens epithelial cells, Notch ligands interact with the Notch receptor. This interaction is necessary for the transcription of *FoxE3* mRNA in lens epithelial cells, which maintains the undifferentiated state, and is located in the embryonic surface ectoderm, leading to the induction of PLE thickening. xSiah2 overexpression did not alter the transcription of *FoxE3* mRNA at st. 24, suggesting that xSiah2 did not affect Notch signaling between retinal progenitor cells and lens epithelial cells. xSiah2 overexpression suppressed the transcription of *Snail-1* and *ESR-1* mRNAs at st. 30. These genes are necessary for the invasion of lens placode through the EMT and the differentiation into lens endothelial cells, which serves as the progenitors for lens fibers, and is located in the anterior portion of the lens between the lens capsule and the lens fibers. Accordingly, xSiah2 might affect Notch signaling in endothelial cells.

## Figures and Tables

**Table 1:** Primers GenBank accession no., cycles, and sequences for PCR.

Factor	Accession no.	Cycles	Sequences
HistoneH4	M21286	24	Forward: 5′-CAGGAGATGGCCACAGCTGC-3′
			Reverse: 5′-TCTTTCTGCATTCTATCAGCA-3′
FoxE3	BC169818	24	Forward: 5′-CCTCTGGAGGCAGGAGAAGA-3′
			Reverse: 5′-TCTGAGGGTTATATCCAGAGCC-3′
β-Crystallin	BC084735	24	Forward: 5′-TGCCTGGAGTGGAACAATGC-3′
			Reverse: 5′-TGTTGAACCATCCCATATGC-3′
Snail-1	BC056857	28	Forward: 5′-CAGCAAGTCTTACTCCACCT-3′
			Reverse: 5′-CTCCTGTGTGTGTTCTGATG-3′
N-cadherin	X57675	27	Forward: 5′-ATGGCGATGGAATTGTATGT-3′
			Reverse: 5′-GAAGACACCGAGTGGAGGTT-3′
ESR-1	AF383157	31	Forward: 5′-ACAAGCAGGAACCCAATGTC-3′
			Reverse: 5′-GCCAGAGCTGATTGTTTGGA-3′

**Table 2:** Primers number and sequences for isolation of xSiah2.

Factor	Primer number	Sequences
xSiah2	Primer 1	GACTAGTGTAATCAATGCCGCCAGAAG
	Primer 2	TAGGATCCTTCGTGTGCCGATTAGGAG
	Primer 3	TACTAGTGCGATGAGCCGCCC
	Primer 4	CGAATTCTTATGGACAACATGTGGAAATGG
	Primer 5	GCGAAGCTTTCAGTGCTGCATAACATTTTC

Underline: restriction enzyme site; double underline: start or stop codon.
